# Generalizability of electrocardiographic artificial intelligence

**DOI:** 10.1038/s44325-025-00078-2

**Published:** 2025-07-15

**Authors:** Ibrahim Karabayir, Oguz Akbilgic

**Affiliations:** Department of Cardiovascular Medicine, Wake Forest School of Medicine, Winston-Salem, NC USA

**Keywords:** Cardiology, Circulation

## Abstract

Electrocardiographic artificial intelligence (ECG-AI) uses AI-based algorithms to analyze ECGs. Recent literature has revealed the untapped potential of ECG beyond traditional diagnostics. ECG-AI is now used not only to detect arrhythmias but also to assess risk and identify both cardiovascular and non-cardiovascular conditions. This perspective article summarizes evidence from published literature to support the conclusion that ECG-AI models are highly generalizable and have the potential to revolutionize healthcare.

## Introduction

Since its discovery by Willem Einthoven in 1902, the electrocardiogram (ECG) has been a cornerstone diagnostic tool for arrhythmias and cardiovascular diseases (CVD), with a robust body of literature defining normal ranges for ECG segment durations and amplitudes^1–5^. Traditionally, ECG interpretation has relied on manual analysis by electrophysiologists, who correlate abnormalities with clinical conditions. However, manual readings can be subjective, influenced by variability in expertise and confounding factors such as medication use. Recent advancements in pattern recognition, neural networks, and high-performance computational hardware (e.g., GPUs, TPUs) have revolutionized ECG analysis, enabling the extraction of abstract features, beyond traditional metrics, correlated with several clinical conditions. These innovations not only enhance diagnostic precision and standardization but also unlock the predictive and prognostic potential of ECG, transforming its role in cardiovascular care.

*Electrocardiographic Artificial Intelligence (ECG-AI)* models are typically implemented in two ways: traditional machine learning based on engineered features or deep learning based on raw ECG. The feature engineering approach involves two steps: Step 1, where ECG features—such as traditional ECG characteristics, heart rate variability metrics, and abstract features generated by signal processing methods or autoencoders—are extracted; and Step 2, where these engineered features are used as inputs to more traditional machine learning methods. In contrast, the deep learning approach directly utilizes raw time-to-voltage ECG data or ECG images as inputs to deep learning models, including convolutional neural networks, long short-term memory networks, recurrent neural networks, graph networks, transformers, and others. Deep learning-based ECG analysis represents a paradigm shift, surpassing traditional ECG analysis methodologies. Numerous studies have demonstrated the superiority of deep learning-based ECG-AI models, primarily due to the complexity and robustness of their architectures, which enable to capture high-dimensional, non-linear patterns that remain inaccessible to traditional ECG feature extraction techniques.

As a low-cost, easily accessible, and remotely applicable tool, ECG-AI offers significant advantages in improving health outcomes, standardizing ECG interpretation, and reducing healthcare resource utilization. However, as any other AI model in healthcare, an ECG-AI model also must possess four key properties: actionability, generalizability, explainability, and trustworthiness^6^. The level of actionability depends on the specific problem being addressed, while explainability can be achieved through post-hoc analyses such as Feature Importance and Direction Analysis^7,8^, Shapley Values^9^, Class Activation Maps^10^, and Attention maps^11^. Trustworthiness, on the other hand, can be viewed as a synthesis of the other three properties. This study focuses on evaluating the generalizability of ECG-AI models from multiple perspectives.

Overfitting is often the primary reason for a lack of generalizability. It refers to excessive tuning of model parameters on the training data, resulting in a model that memorizes the input-output relationship rather than learning it. This type of overfitting can be mitigated through rigorous cross-validation strategies and evaluated using an internal holdout test set or independent validation sets. However, specific to ECG-AI applications in healthcare, overfitting can also occur when models are overly tailored to data from a specific population or certain ECG devices and software. This perspective paper focuses on the generalizability of ECG-AI models.

## Methods

This Perspective paper discusses the generalizability of ECG-AI from six different perspectives based on several studies from published literature presented as two case studies for heart failure (HF) risk prediction and HF detection. The results in these case studies bring together results of ECG-AI models when tested on different demographics, cohort studies, as well as reporting results for ECGs retrieved from different cardiology information systems, including MUSE (GE Healthcare) and EPIPHANY (Baxter, Inc.).

### Case Study 1: ECG-AI for 10-year incident HF risk prediction

Over 6.5 million people in the United States are living with HF^12^. Identifying individuals at risk for HF is crucial for implementing preventive care interventions that can help reduce the burden of the disease. Traditional HF risk calculators use well-known HF risk factors as inputs. Examples include the Framingham Heart Study’s HF risk calculator (FHS-HF)^13^ and the Atherosclerosis Risk in Communities study’s HF risk calculator (ARIC-HF)^14^, which were developed using data from the FHS^15^ and ARIC^16^ cohort studies. Additionally, an ECG-AI model has been developed to predict the 10-year risk of incident HF using a convolutional neural network architecture based solely on 12-lead, 10-second ECG data. This model was trained on 80% of ARIC data and tested on the remaining 20% as a holdout set^17^. It was further externally validated using data from the Multi-Ethnic Study of Atherosclerosis (MESA)^18^, and real-world data from the University of Tennessee Health Science Center (UTHSC), Memphis, TN^19,20^. Table 1 below summarizes the results of the FHS-HF, ARIC-HF, and ECG-AI-HF models for 10-year incident HF risk prediction on the same datasets.

**Table 1:** 10-year incident HF Risk Prediction

Data source		ARIC data	MESA data	UTHSC data
Data Type		Cohort Study	Cohort Study	Real World/EHR
Demographics	Sex	45% male	47% male	55% male
	Race	64% White 36% Black	39% White 28% Black 12% Chinese American 22% Hispanic	42% White 54% Black
	Age (years)	54 ± 6	62 ± 10	64 ± 13
ECG Data Acquisition Dates		1987–1989	2000–2002	2008–2020
10-year incident HF rate		6%	4%	26%
ECG source	Vendor	GE MUSE	GE MUSE	EPIPHANY
10-year HF prediction AUCs	ECG-AI* (input: ECG alone)	0.76 (0.72–0.80)	0.77 (0.74–0.79)	0.77 (0.76–0.79)
	FHS-HF Model** (inputs: Age, BMI, heart rate, systolic blood pressure, diabetes mellitus, coronary heart disease, left ventricular)	0.78 (0.74–0.83)	0.74 (0.70–0.78)	0.72 (0.70–0.74)
	ARIC-HF Model*** (inputs: Age, sex, race, BMI, heart rate, systolic blood pressure, diabetes mellitus, smoking status, hypertension)	0.80 (0.75–0.85)	0.76 (0.72–0.80)	0.71 (0.69–0.73)

* ECG-AI is a CNN model developed on 80% of ARIC data using waveform 12 lead ECG as the only input.

** FHS’s HF Model is a pooled logistic regression model. The original FHS model, without any modification, was applied to a same ARIC hold out, MESA, and UTHSC data.

*** ARIC Study’s HF Model is a Cox Regression model with input variables of. The original FHS model, without any modification, was applied to a same ARIC hold out, MESA, and UTHSC data.

### Case Study 2: Detection of left ventricular dysfunction (LVD) and heart failure with preserved ejection fraction (HFpEF)

HF diagnosis traditionally relies on clinical history, physical examination, blood biomarkers, and imaging modalities such as echocardiography and cardiac MRI, the gold standards for detecting left ventricular dysfunction (LVD). However, these techniques are costly, not routinely performed, and often result in missed or delayed diagnoses, particularly in asymptomatic LVD cases where imaging is not pursued due to ambiguous symptoms. Furthermore, imaging alone is insufficient for diagnosing heart failure with preserved ejection fraction (HFpEF), as ejection fraction levels remain within the normal range. A recently developed ECG-AI model enables simultaneous classification of reduced ejection fraction (rEF; LVEF < 40), mid-range ejection fraction (mEF; 40 ≤ LVEF < 50), and HFpEF (LVEF ≥ 50 with HF) from 12-lead ECG data^21^. A single-lead, lead I, variant demonstrated comparable performance. Trained on over 1 million ECGs from 165,243 participants at Atrium Health Wake Forest Baptist (AHWFB), the model was externally validated on adult and pediatric cohorts from the UTHSC. Table 2 highlights cohort characteristics and the performance of models. These findings demonstrate the potential of multi-class ECG-AI models, leveraging 12-lead and single-lead ECG data, as a scalable, cost-effective solution for HF subtype prescreening, enabling earlier detection and more efficient allocation of healthcare resources.

**Table 2:** ECG-AI for simultaneous detection of rEF, mEF, and HFpEF

Data Source			WFBH-Adult	UTHSC-Adult	UTHSC-Children
Data Purpose			Derivation/Internal Validation	External Validation	External Validation
Demographics	Male	Controls	63,756 (46)	17,447 (42)	4093 (49)
		rEF	5513 (64)	332 (65)	63 (88)
		mEF	8116 (62)	273 (64)	8 (18)
		HFpEF	1687 (46)	232 (42)	18 (49)
	Black	Controls	26,148 (19)	24,559 (59)	3354 (41)
		rEF	1775 (21)	305 (59)	7 (12)
		mEF	2495 (19)	207 (50)	42 (93)
		HFpEF	749 (20)	304 (54)	23 (62)
	Age at ECG	Controls	57.44±15.11	53.48±17.85	8.71±6.09
		rEF	65.68±14.7	62.69±16.21	12.84±3.62
		mEF	64.36±15.39	63.53±15.61	5.02±5.54
		HFpEF	66.82±13.89	66.42±14.82	7.50±7.53
Outcome		Control	993,202 (92)	69,439 (95)	8,276 (98)
		rEF	32,962 (4)	1320 (2)	60 (1)
		mEF	40,997 (3)	707 (1)	45 (0.5)
		HFpEF	11,037 (1)	1336 (2)	37 (0.5)
ECG source		Vendor	GE MUSE	EPIPHANY	EPIPHANY
12 Lead Model AUCs		rEF	0.90 [0.89–0.91]	0.92 [0.91–0.92]	0.97 [0.96–0.99]
		mEF	0.81 [0.80–0.83]	0.76 [0.75–0.77]	0.71 [0.65–0.79]
		HFpEF	0.80 [0.78–0.82]	0.73 [0.72–0.74]	0.64 [0.56–0.74]
Lead I Model AUCs		rEF	0.89 [0.88–0.90]	0.90 [0.90–0.91]	0.94 [0.91–0.95]
		mEF	0.78 [0.76–0.79]	0.75 [0.74–0.77]	0.77 [0.71–0.84]
		HFpEF	0.75 [0.73–0.77]	0.74 [0.73–0.75]	0.67 [0.58–0.74]

Values are presented as *N* (%) for categorical variables and Mean ± SD for continuous variables.

## Results

The results from Use Case 1 and Use Case 2 are summarized below under six subsections.

### Generalizability from one population to another

As presented in Table 1, The ECG-AI model for predicting 10-year incident HF was developed using data from the ARIC study, which included only White and Black American participants. Despite this, the model demonstrated similar accuracy (DeLong Test, *p* = 0.616) when applied to both the ARIC and MESA cohorts, even though the MESA cohort also included Chinese American and Hispanic participants. Moreover, the MESA validation cohort was significantly older than the ARIC derivation cohort, indicating that the ECG-AI model is generalizable across populations with varying racial and age profiles.

A similar trend was observed with another ECG-AI model developed for incident LVD and HFpEF detection model in Table 2^21^. A 12-lead ECG-based ECG-AI model was trained using data from WFBH and externally validated on data from UTHSC with comparable performance. The accuracy of rEF detection was not reduced in UTHSC external validation data, however, the accuracy of mEF and HFpEF detection was slightly but statistically significantly reduced (DeLong Test, *p* < 0.01 and *p* < 0.001 for mEF, and HFpEF, respectively).

Table 1 indicates that traditional clinical risk factor-based models, such as FHS-HF and ARIC-HF, do not exhibit the same level of generalizability as the ECG-AI model. The ARIC-HF model, which was originally developed using ARIC data and achieved an AUC of 0.80, saw its AUC decrease to 0.76 when applied to MESA data (DeLong test, *p* = 0.08). A similar trend was observed with the FHS-HF model, which was developed primarily using data from White Americans; its AUC dropped from 0.80 in ARIC data to 0.76 in MESA data (DeLong test, *p* = 0.08). This decline in performance is expected in models driven by clinical risk factors, as the prevalence of these inputs can vary significantly between populations, leading to bias toward the derivation cohort. For instance, hypertension, a known risk factor for HF, has varying prevalence, treatment adherence, and response across different racial groups^22^. Consequently, the impact of hypertension on HF risk may differ from one population to another based on cohort characteristics. Thus, traditional risk calculators that rely on conventional risk factors should be trained on cohorts’ representative of the intended population for implementation. In contrast, ECG-AI models, which leverage only ECG data, demonstrate broader generalizability across diverse populations.

Despite the promising results summarized here about generalizability across cohorts, there is still a need for further information on the broader generalizability, assessing how an ECG-AI model developed in one country would be applicable to data from another country with significantly different racial, ethnic, cultural, and geographic differences. Therefore, incorporation of simple demographic variables with ECG data can result in not only more generalizable but also more accurate ECG-AI models.

### Generalizability from cohort studies to real world data

The development of ECG-AI models using retrospective data is generally limited to individuals who have had ECGs recorded. However, ECGs are not commonly part of routine annual screening exams. Consequently, ECGs available in electronic health records (EHR) reflect a biased cohort consisting of individuals who required an ECG for clinical reasons. Therefore, it is necessary to demonstrate the broader generalizability of EHR-driven ECG-AI models for population-level screening purposes. This can be achieved through prospective studies or by testing the models on cohort studies where ECGs were recorded as part of the study protocol rather than for clinical indications. A similar question of generalizability arises when an ECG-AI model is developed using data from cohort studies, raising concerns about its applicability to real-world clinical data.

Table 1 shows that the ECG-AI model, developed and validated using data from the ARIC and MESA cohort studies, maintains almost identical accuracy when tested on real-world EHR data from UTHSC (DeLong Test, *p* = 0.572), despite substantial differences in cohort characteristics. In contrast, the accuracy of traditional HF risk calculators, such as FHS-HF and ARIC-HF, significantly declined when applied to real-world data (DeLong test, *p* < 0.001 for all comparisons). This may be attributed not only to the notable differences in cohort characteristics between ARIC and UTHSC, as previously discussed, but also to the potential unreliability of EHR-driven risk factors due to miscoded ICDs or underdiagnosed conditions within EHRs.

In summary, the results in Table 1 indicate that the ECG-AI model demonstrates generalizability between cohort studies and real-world data, whereas traditional risk calculators lack generalizability when applied to real-world data.

### Generalizability across ECG vendors

Resting 12-lead, 10-second ECGs are retrieved, analyzed, and presented by various cardiology information systems from different vendors operating in the United States. Each vendor typically employs proprietary processing and filtering algorithms to handle raw voltage data from patients. Therefore, the vendor-to-vendor generalizability of ECG-AI models needs to be assessed. Both Tables 1 and 2 demonstrates that the ECG-AI model, developed using data from the GE MUSE system, was successfully validated on data from another cardiology information system, EPIPHANY, at UTHSC. This pattern was also observed in other studies focused on HF detection^23^, Parkinson’s Disease prediction^24–27^, preeclampsia prediction^28^, and peripartum cardiomyopathy detection^29^, where ECG-AI models developed using GE MUSE-derived ECGs were successfully validated with EPIPHANY-derived ECG data.

There is additional evidence in the literature suggesting that clinical 12-lead ECG-driven models for low ejection fraction (EF) detection have been successfully adapted to ECGs recorded from ECG-enabled stethoscopes^30^. Moreover, studies indicate concordance in risk predictions between lead I of clinical 12-lead ECGs and Apple Watch ECGs for an ECG-AI model designed to predict fatal coronary heart disease^31^. However, the generalizability of clinical ECG-driven ECG-AI models to wearable ECGs remains an area requiring further investigation, primarily due to significant differences in data collection techniques, as well as the electrodes and sensors used.

### Generalizability from old ECGs to new ECGs

AI now facilitates automated and comprehensive analysis of medical images in ways beyond human visual capacity, leading to enhanced diagnostic and predictive performance, especially when using advanced imaging technologies such as magnetic resonance imaging (MRI), computed tomography (CT), and echocardiograms. However, despite their detailed granularity compared to surface ECGs, these imaging technologies evolve rapidly. Consequently, the longitudinal validity of AI models based on such advanced imaging, even if highly accurate, remains uncertain due to continuously changing image quality. In contrast, while advances in ECG technology may change the methods of recording, they do not significantly alter the raw ECG data itself. This enables ECG-AI models to leverage billions of retrospectively available ECGs within healthcare systems to develop disease detection and risk prediction models. This advantage is also illustrated by our results in Table 1, which show that the HF risk prediction ECG-AI model, developed using data recorded between 1987–1989, maintained the same level of accuracy when applied to ECGs recorded up to 2020. Such time-invariant generalizability is one of the most significant strengths of ECG-AI models compared to AI models based on other imaging modalities.

### Generalizability from Adults to Children

Children are underrepresented in AI literature, as well as in ECG-AI literature. For example, a PubMed search using keywords of “Artificial Intelligence” and “Adult” returns 26,993 publications between January 1st, 2020, and May 4th, 2025, while there are only 9,924 results are returned for the same data range when keywords of “Artificial Intelligence” and “Children” were used. Similarly, for the same data range, PubMed search for “Artificial Intelligence” and “Electrocardiogram” and “Adult” keywords returns 517 results, while “Artificial Intelligence” and “Electrocardiogram” and “Children” keywords return only 90 results. Such underrepresentation may stem from many reasons. First, age is one of the common risk factors for CVD. A study of over 20 million Americans showed, for example, that the average age (SD) at first onset of major CVD, including ischemic heart disease, heart failure, and strok,e is 54 (16) years^32^. Other than major CVD being rare in children, another contributing factor why adult-driven ECG-AI models are not typically applied to children may be that children’s hearts differ structurally and physiologically from adults, as do key ECG characteristics, necessitating evaluation of ECG-AI model generalizability across age groups. Nevertheless, despite it may be rare, CVD occurs in children too. And despite the structure of the heart and well-known ECG characteristics may be different for adults and children, it is still and, despite children and adult hearts may differ significantly, ECG-AI models trained to classify LVD and HFpEF in adults were tested on pediatric data (Table 2). The 12-lead model showed strong accuracy for detecting low EF (EF < 40%), with an AUC of 0.92 in adults and 0.97 in children, but performance declined for mid-range EF (AUC 0.76 to 0.71) and HFpEF (AUC 0.73 to 0.64). The Lead-I model demonstrated better generalization: rEF (0.90 to 0.94), mEF (0.75 to 0.77), and HFpEF (0.74 to 0.67). Variations in HF subtype prevalence, extreme rareness of HFpEF in children, and ECG differences likely contributed to these discrepancies. While generalization for rEF was notable in both models, pediatric cohort size was limited, underscoring the need for larger studies and potentially pediatric-specific ECG-AI models.

Despite the results from one study mentioned here, future work is needed to test the generalizability of ECG-AI models from adults to children.

### Generalizability of 12-Lead vs. Single-Lead ECG-AI Models

Recent research comparing the performance of 12-lead and single-lead (Lead I) ECG-AI models for detecting LVD and HFpEF revealed notable insights into their generalizability^21^. Both models were developed using identical derivation cohorts, architectures, and hyperparameters, differing only in input data. In internal and external validation cohorts, the 12-lead model achieved AUCs of 0.81 and 0.76 for moderately reduced ejection fraction (mEF) and 0.80 and 0.73 for HFpEF, respectively (DeLong *p* < 0.01). The Lead I model, by comparison, recorded AUCs of 0.78 and 0.75 for mEF and 0.75 and 0.74 for HFpEF (DeLong *p* > 0.01), demonstrating consistent predictive performance across cohorts (Table 2).

The robustness of the simpler Lead I model may enhance its generalizability, particularly in diverse settings and populations. This stems from the simplistic and straightforward nature of Lead I data collection, which involves electrode placement between the left and right arms. Unlike multi-lead setups, Lead I is less prone to misplacement errors – reported in up to 64% of cases^33^, such as lead reversals, which can produce pseudo-abnormalities. Such errors are more common among less experienced technicians, contributing to variability in 12-lead recordings.

Since all ECG leads measure the same electrical activity of the heart from different angles, a single-lead approach like Lead I may serve as a “pure” and standardized data source, reducing variability and enhancing the model’s robustness. This is particularly advantageous when the chosen lead adequately captures the relevant signal for the targeted cardiovascular condition. In summary, while 12-lead ECG-AI models can leverage more comprehensive data for enhanced diagnostic performance, the simplicity and stability of single-lead models may make them more generalizable and suitable for broader implementation.

The overarching goal of ECG-AI analysis would indeed be to translate these models into single-lead ECGs recorded from wearables such as a smartwatch with single-lead ECG functionality. A recent article found that a single-lead ECG-based ECG-AI model for risk prediction of fatal coronary heart disease provided highly concordant results when the lead of the 12-lead clinical vs single-lead Apple Watch ECGs were used^34^. There are billions of clinical ECGs linked to patient-level data in our healthcare systems to support the development of generalizable ECG-AI models. However, it is not the case for smartwatch ECGs since they are relatively new technologies, while such data is extremely rarely captured and linked to patient-level clinical data. Hence, developing ECG-AI models using lead I of these large clinical ECG repositories, then fine-tuning them in relatively small wearable ECG repositories and implementing them in HIPAA-compliant platforms such as ECG-Air^35^ can lead to significant improvements in remote patient monitoring and telemedicine.

## Conclusions

When developed and designed carefully, ECG-AI models can be time-invariant and generalizable across ECG vendors and patient populations with varying race, sex, and age. The generalizability of ECG-AI models holds great promise for fostering trustworthy AI. The integration of ECG-AI models into healthcare systems and individual practices can facilitate the timely detection of various health conditions, enabling providers to implement early interventions. Due to its reliance on low-cost and widely accessible ECG data, as well as its strong generalizability, ECG-AI has the potential to reduce healthcare disparities.

Advances in biomedical engineering have resulted in more wearables with FDA-cleared ECG functionality. However, existing use of these wearable-generated ECGs is mostly limited to atrial fibrillation detection using more traditional ECG analysis. At this point, the critical need is to connect the ECG collected from these devices with patient health records. Thereby, large wearable ECG repositories can be created and linked to certain conditions and disease states. This way, ECG-AI model implementation would not be restricted to device manufacturers by allowing research institutes to develop and implement their own ECG-AI algorithms customized to any outcome of the patient population of interest. Despite such an approach may present bias in healthcare due to access to such smart wearable devices, their proven utility in reducing healthcare burden would further initiate the distribution of such devices by insurance companies and healthcare systems at no cost to individuals.

## Data Availability

No datasets were generated or analyzed during the current study.
